# Effect of the Antibiotic Neomycin on the Toxicity of the Glycoside Vicine in Rats

**DOI:** 10.1155/2013/913128

**Published:** 2013-06-12

**Authors:** Mahmoud S. Arbid, Khaled M. M. Koriem, Gihan F. Asaad, Hoda A. Megahed

**Affiliations:** ^1^Department of Pharmacology, National Research Centre, El Buhouth Street, Dokki, Giza 2311, Egypt; ^2^Department of Medical Physiology, National Research Centre, El Buhouth Street, Dokki, Giza 2311, Egypt; ^3^Integrative Medicine Cluster, Advanced Medical and Dental Institute (AMDI), Universiti Sains Malaysia (USM), No. 6 Tingkat 1, Persiaran Seksyen 4/9 Bandar Putra Bertam, 13200 Kepala Batas, Pulau Pinang, Malaysia; ^4^Department of Medical Biochemistry, National Research Centre, El Buhouth Street, Dokki, Giza 2311, Egypt

## Abstract

Vicine is hydrolyzed by microflora to highly reactive free radical generating compound divicine which causes mortality and other adverse effects. This study in the rats established the effect of a broad spectrum and poorly absorbed antibiotic, neomycin sulfate on the toxicity of vicine. The results showed extremely decrease in mortality rate in the group pretreated with neomycin. Hemoglobin (Hb) concentration, hematocrit (Hct) value, and red blood cells (RBCs) count were significantly decreased after injection of vicine and the improvement of these values in the group pretreated with neomycin. The same results were observed in white blood cells (WBCs). The results showed a significant decrease in glucose level and returned to normal in group pretreated with neomycin. Glutathione (GSH) was significantly decreased in the vicine group and returned to normal value in the group pretreated with neomycin. Lipid peroxide (TBARs) was significantly increased in the group treated with vicine and neomycin pretreated group decreased to the normal level. Glucose-6-phosphate dehydrogenase (G6-PD) activity was significantly decreased and returned to normal level in rats pretreated with neomycin. Serum protein and globulin were significantly decreased but serum albumin showed insignificant decrease in vicine and neomycin groups compared to control. Alanine aminotransferase (ALT) and aspartate aminotransferase (AST) were significantly decreased in the vicine group. The group pretreated with neomycin showed significantly increased activities of AST and ALT compared with vicine group. In conclusion, neomycin pretreatment of rats injected with glycoside vicine decreased to a great extent of its toxic and mortality effects and is useful in favism and hemolytic anemia.

## 1. Introduction

The importance of legumes in agriculture, human consumption, and animal nutrition is increasing exponentially due to the increasing world population and its need for proteins. Food legumes are considered the best substitute for meat in many parts of the world, where there is a demand for alternate, nonanimal protein sources. Legume crops have two distinctive traits: (1) their high protein content, and (2) their unique symbiotic ability to fix atmospheric nitrogen in the soil. Faba bean (*Vicia faba* L.) is an important member of the legume family with highly useful characteristics. The world production of faba beans is close to 4.5 millions of tons. Faba bean is the second most important legume crop in Europe, which accounts for 14% of the world area and about 25% of the world production [1]. It is widely grown and consumed, especially in Egypt, Mediterranean region, China, North African countries and parts of Europe, and South America, and is served in a great variety of forms, mostly based on the immature or mature seed. For both humans and livestock, it provides high-quality, lysine-rich proteins, carbohydrates, and fibers. It is also rich in carotenoids, vitamins [2, 3], and essential minerals including iron, magnesium, potassium, zinc, copper, and selenium. Faba beans have also lipid-lowering effects and may also be a good source of antioxidants and chemopreventive factors [4]. 

In common with numerous crop legumes, faba bean produces various antinutritional factors including raffinose series oligosaccharides, lectins and protease inhibitors, phytate, and tannins. Almost unique to faba bean are vicine and convicine, the causative agents of favism in many human populations. These antinutritional factors have limited its worldwide acceptance as a competitive food crop. As an effective nitrogen-fixing species, it is regarded as an excellent crop for soil amendment, which also provides high-quality fodder and silage. Elsewhere around the world, the crop is very widely distributed in the Mediterranean region, the Nile valley, Ethiopia, Central Asia, and Northern Europe. Faba bean is grown as a seed crop mostly along the west coast of the United States. In South America, faba bean is also of importance as a food crop especially in the Andean region. Ray and Georges [5] detected convicilin, oleosin, fabatin, and defensin in faba beans seeds. On the other hand, Bicakci [6] reported blood in the urine, headache, dizziness, fatigue, loss of appetite, and jaundice in the eyes 24 hours after eating large amounts of fresh faba beans seeds. Laboratory investigation revealed hemolytic anemia, hyperbilirubinemia, and G6-PD deficiency. He detected that approximately 0.5% of fava bean seeds have 2 pyrimidine beta-glycosides called vicine and convicine. Faba bean has 0.73% vicine, 0.08% convicine, and 0.53% beta-cyanoalanine glycosides. Furthermore, Gutierrez et al. [7] used two cleavage amplified polymorphism (CAP) markers in faba beans breeding to track the introgression of the vicine and convicine allele to develop cultivars with low vicine and convicine contents and improved crop nutritional value. 

Vicine is a glycoside that is found primarily in Faba beans (*Vicia faba* L.) which is one of the most important pulse crops in the world, being consumed in large quantities in the Middle East and North Africa particularly Egypt [8]. Vicine is a compound which is hydrolysed by intestinal microflora to high reactive free radical generating compound divicine [9]. Divicine has been strongly implicated as the causative agent in favism (*Vicia faba* anemia) [10], a hemolytic disease in humans particularly young males that have deficiency of erythrocytic glucose-6-phosphate dehydrogenase (G6-PD) activity [11]. These free radical generators may also cause other adverse effects including lipid peroxidation [12] and altered fat [13]. In broiler chicken, reduction of vicine and convicine contents significantly increases the apparent metabolizable energy value [14]. Moreover, Farran et al. [15] stated that high levels of vicine or convicine or both might have shortened birds' survival time by enhancing the neurotoxicity induced by lower levels of total beta-cyanoalanine (BCA).

Neomycin sulphate is a broad spectrum and poorly absorbed antibiotic which inhibits the growth of anaerobic microorganisms in gastrointestinal tract. In addition to its antibiotic effect, it has other effects, for example, inhibition of rat glioma growth [16], and it has a significantly higher rate of wound closure [17]. On the other hand, Sun et al. [18] proved that neomycin reduced the intracellular calcium response in osteoblasts by 27%. This inhibitory effect of neomycin was more pronounced (75% reduction in maximum fluorescence) for osteoblasts seeded on notched cortical bone. Moreover, neomycin in conjugation with polyhexanid and propamidinisoethionat used for treatment of acanthamoeba keratitis disease and acanthamoeba keratitis patient often heals appropriately [19]. Furthermore, Neomycin blocks aminoacyl-transfer RNA (aa-tRNA) selection and translocation as well as ribosome recycling by binding to helix 69 (H69) of 23S ribosomal RNA within the large subunit of the *Escherichia coli* ribosome [20]. 

The purpose of this study was providing evidence to the effect of orally administered antibiotic neomycin on the toxicity of vicine.

## 2. Materials and Methods

### 2.1. Animals

Male albino rats (100 ± 5 g) were obtained from the animal house of the National Research Centre (Dokki, Giza, Egypt) and were kept in special plastic cages. The animals were maintained on commercial balanced diet and tap water. The experiments were performed after approval from the ethics committee of the National Research Centre and in accordance with recommendations for the proper care and use laboratory animals (NIH publication no 85:23 revised 1985).

### 2.2. Materials

Vicine was prepared as described by Marquardt et al. [21]. Neomycin sulphate tablets (500 mg each) were purchased from Memphis Co. (Cairo, Egypt). All diagnostic kits used were of analytical grade and were obtained from Gamma Trade Co., Egypt.

### 2.3. Experimental Design

#### 2.3.1. First Experiment

*Determination of Mortality Rate of Vicine and Neomycin*. A total number of 50 rats were used in this experiment and the rats were divided into 5 equal groups (10 rats/group) and treated as follows:

- control: injected intraperitoneally (ip) with 1 mL physiological saline,
- groups 2, 3 and 4: injected ip with vicine in a dose of 100, 200 and 400 mg/kg bwt, respectively,
- group 5: administered orally 4 times with neomycin (250 mg/kg bwt) a day prior to the administration of 400 mg/kg bwt vicine ip.

Then, throughout 48 hours experimental period, the total number of died and survived rats was recorded.

#### 2.3.2. Second Experiment

Based on the result of the first experiment, a total number of 24 rats were used in the second study and the animals were divided into 3 equal groups each of 8 animals as follows:

- control group: injected ip with 1 mL physiological saline once a day over 7 days period,
- vicine group: injected ip with vicine in a dose of 400 mg/kg bwt once a day over 7 days period,
- Neomycin + Vicine group: administered orally 4 times 250 mg/kg bwt neomycin a day prior to the administration of vicine (400 mg/kg bwt, ip) once a day over 7-day period.

### 2.4. Blood Sampling and Handling

At the end of 7 days experimental study, the blood samples were collected before rats being scarified from retroorbital plexus of rats using capillary tubes into clean centrifuge tubes. Part of blood samples was collected using EDTA as an anticoagulant for hematological parameters and erythrocyte glutathione. The other part of the blood sample was allowed to coagulate and centrifuged at 4000 rpm for 15 min to separate blood serum. Separated serum was stored at −20°C for the determination of the other parameters and the following parameters were done.

*Hematological Parameters*. Red blood cells (RBCs), white blood cells (WBCs) count, and hematocrit, value were carried out according to the method of Rodak [22]. Hemoglobin (Hb) concentration was determined by the method described by Van Kampen and Zijlstra [23].

*(1) Determination of Reduced Glutathione (GSH)*. Erythrocyte glutathione concentration was determined by the method described by Beutler et al. [24].

*(2) Determination of Serum Albumin Content*. Albumin content in serum was estimated according to the method described by Henry [25].

*(3) Determination of Serum Globulin Content*. Serum globulin content was calculated by subtracting the individual data of serum albumin from individual data of serum total protein.

*(4) Determination of Transaminases Activities (AST & ALT).* Aspartate and alanine transaminases (AST & ALT) activities in serum and liver tissue were estimated colorimetrically according to the method described by Reitman and Frankel [26].

*(5) Determination of Serum Glucose Level*. Serum glucose was measured according to the enzymatic colorimetric method described by Trinder [27].

*(6) Estimation of Lipid Peroxidation Product (Malondialdehyde, MDA)*. Lipid peroxidation is estimated in serum according to the method described by Yoshioka et al. [28].

*(7) Determination of Glucose-6-Phosphate Dehydrogenase (G6-PD). *G6-PD activity in serum was estimated according to the method of Loher and Waller [29].

### 2.5. Liver Tissue Preparation

After collection of the blood, the animals were decapitated and then dissected, whereby the liver was obtained, washed in cold saline, and dried between filter papers. The liver was weighed, homogenized, and kept at −20°C for further investigation; 0.5 gm of liver tissue was dissolved in 2.5 mL of Tris buffer solution, then homogenized in the Homogenizer for exactly 30 min. Then, it was centrifuged for exactly 20 min at 7000 rpm, which separated the supernatant, which proceeded in the same manner of blood serum for the determination of liver transaminases and protein.

### 2.6. Histopathological Examinations

Specimens of liver were fixed at 10% neutral formalin solution and then processed for routine embedding in paraffin. Blocks were sectioned at a thickness of 5 *μ*m and stained with hematoxylin and eosin for histopathological examination. 

### 2.7. Statistical Analysis

Results were expressed as mean ± standard deviation (SD). Differences between groups were assessed by ANOVA using the SPSS 13 software package for Windows. Post hoc testing was performed for intergroup comparisons using the least significant difference (Tukey) test, significance at *P* values ≤ 0.05.

## 3. Results

**Figure 1:**
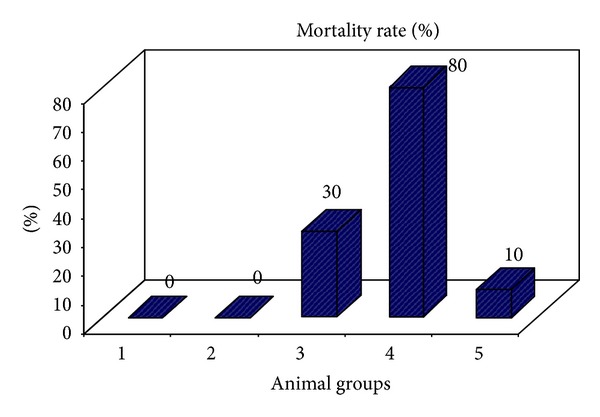
Mortality rate percentage of rats injected with vicine and animals pretreated with antibiotic neomycin to vicine-injected rats.

The first experiment demonstrated that vicine alone at dose of 100 mg/kg body weight (bwt) did not cause any mortality. Mortality started at the dose of 200 mg/kg bwt and all the rats died at the dose of 400 mg/kg bwt. In the fifth group which is administered neomycin 250 mg/kg bwt 4 times a day prior to administration of vicine (400 mg/kg bwt), only one from 10 rats died (Table 1 and Figure 1).

The results of the second experiment demonstrated that there were significant decrease in Hb concentration in vicine-induced group as compared to control group meanwhile the rats treated with neomycin prior to vicine showed improvement in Hb. Concerning Hct value (%), the results indicated that the vicine group exhibited a significant decrease as compared with control group. This decrease showed an improvement in the group treated by neomycin prior to vicine. RBCs count exhibited similar behavior as Hb concentration and Hct value which showed a very highly significant decrease in vicine group and marked improvement was observed in the group receiving neomycin prior to vicine as compared to the mean control value. On the other hand, the mean value of WBCs count showed insignificant decrease in vicine group. Rats that received neomycin prior to vicine showed an increase in WBCs count (Table 2).

Table 3 showed that the mean level of serum glucose significantly decreased in vicine group but in the group that received neomycin prior to vicine the results were almost around the control values. Concerning the concentration of GSH in blood, results revealed a highly significant decrease in vicine group. The decrease in blood GSH concentration in blood was improved in the group that received neomycin prior to vicine. TBARs level in liver tissue exhibited a very highly significant increase in the group treated with vicine but in the group treated with neomycin prior to vicine the TBARS level approaches the normal level. The mean value of the activity of G6-PD in serum is illustrated in Table 3. The results showed a highly significant decrease in G6-PD activity in vicine group compared to the control value. This decrease was restored to be almost near the control value in rats treated with neomycin prior to vicine.

In Table 4, serum total protein content showed a significant decrease in vicine compared to control and in neomycin treated groups the globulin and protein tend to be normal values. The serum albumin showed a trend almost around the control level in vicine group and in the group received neomycin prior to vicine. Concerning serum globulin results showed a highly significant decrease in vicine group and this will be improved in the group receiving neomycin prior to vicine. Total protein content in liver tissue showed significant decrease in vicine group while when neomycin administered prior to vicine an increase in liver total protein content was observed compared to vicine group.

The effects of vicine and neomycin prior to vicine on AST and ALT activities in serum of male albino rats were done; the results were demonstrated in Table 5. In vicine group, there was very highly significant decrease in serum AST activity meanwhile in the group treated with neomycin prior to vicine the results showed an increase in serum AST if compared with vicine group. Serum ALT showed significant decrease in serum ALT activity meanwhile in the group treated with neomycin prior to vicine the results showed an increase in serum AST compared with vicine group. The mean liver AST activity showed a very highly significant increase in vicine group. This increase became lesser in the group that received neomycin prior to vicine. ALT activity in liver tissue exhibited a similar behavior as that observed in liver AST as compared to the control group and neomycin prior to vicine decreased liver ALT to approach the control value.

**Figure 2:**
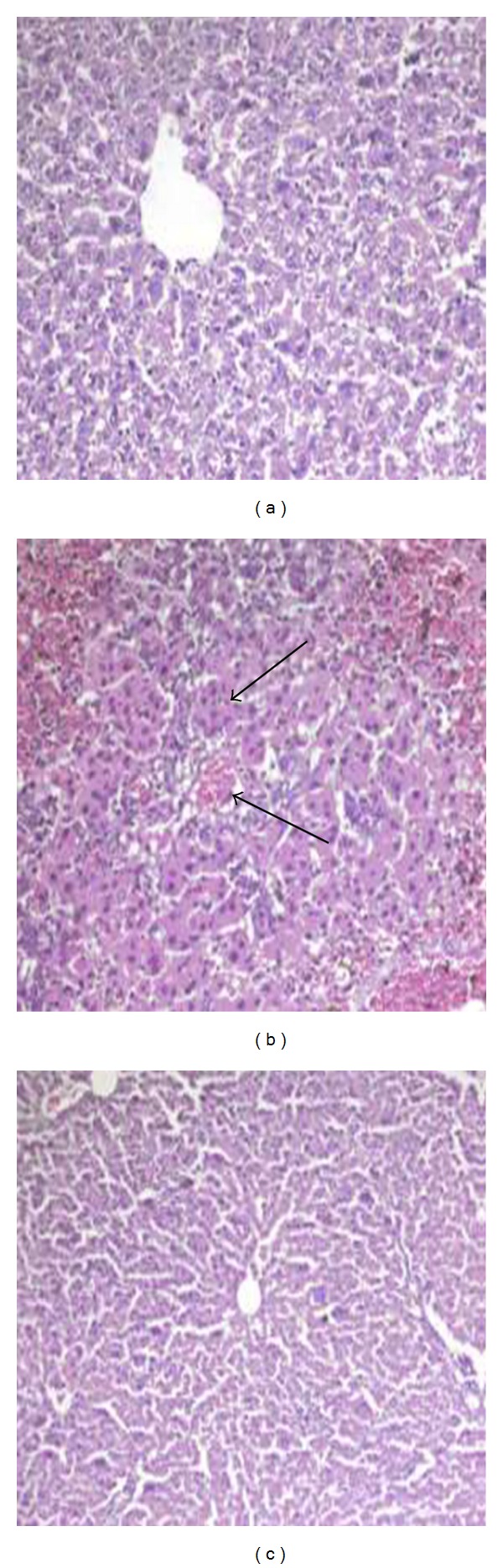
(a) shows the control group with preserved hepatic architecture (H&EX200). (b) shows vicine treated group with lost hepatic lobular architecture, large areas of hemorrhages in the fibrous strands, and cirrhosis (H&EX200). (c) shows pretreatment of antibiotic neomycin to vicine-treated rats with preserved hepatic lobular architecture. The hepatocytes are within normal limits and preserved their plate pattern. Liver almost returns to the normal pattern (H&EX200).

Figure 2 revealed the histopathology results in vicine and neomycin pretreated groups. The structure of the control liver showed normal hepatocytes, vascular sinusoids, and centrolobular vein (Figure 2(a)). Injection of vicine to rats showed losses of hepatic lobular architecture, with large areas of hemorrhages in the fibrous strands between hepatic nodules and liver cirrhosis (Figure 2(b)). Examination of liver sections of rats pretreated with neomycin prior to vicine showed preserved hepatic lobular architecture. The hepatocytes were within normal limits and preserved their plate pattern. Liver almost returned to the normal pattern (Figure 2(c)).

## 4. Discussion

The glycoside vicine that is found in the faba beans (*Vicia faba* L.) when administered intraperitoneally at relatively large amounts resulted in a rapid decrease in the concentration of blood GSH followed by death of the animals, where 400 mg/kg vicine was responsible for 80% mortality rate after 48 hours and no more deathes were observed in rats when administrated vicine for 7 days while 500 mg/kg vicine gives mortality rate of 100% during 7-day treatment [11]. These results can be attributed to the rapid uptake and hydrolysis of this glycoside by the intestinal microflora [9, 30], to highly reactivate free radical generating compound divicine which has been strongly suggested to be the causative agent in favism [31]. Divicine when absorbed in sufficient quantity into the blood provides evidence that this compound is toxic. Vicine must be chosen in high concentration because vicine first converted to its aglycone (divicine) to become biologically active and this conversion needs high vicine quantities or alternatively the process is sufficiently slow so that the animal is able to neutralize most of the products of divicine that are formed following the hydrolysis of vicine [11]. Single injection of vicine caused a decrease in blood glutathione concentration followed by hemolysis and hemoglobin changed from oxyhemoglobin to methemoglobin so that animals appeared to die of asphyxiation [11].

The mechanism of neomycin protection was based on the hypothesis that vicine was hydrolysed by intestinal microflora [30] to produce divicine, where neomycin produced morphological changes in intestinal microflora [32] to prevent the hydrolysis of vicine to its aglycone (divicine) which is the causative agent of favism (*Vicia faba* anemia).

This study conducted the previous preliminary study of Arbid et al. [33], which demonstrated that neomycin reduced the rate at which vicine and convicine were hydrolysed in the gastrointestinal tract, and the neomycin reduced toxicity of both, while this study investigated that neomycin prevents the hydrolysis of vicine to its aglycone (divicine) and protects against the oxidative effect of vicine and this antioxidant effect of neomycin was confirmed by measuring hematological parameters, serum, and liver proteins and transaminases, in addition to liver histopathology and this can be useful in protection in case of favism and hemolytic anemia. 

In the present study, the effect of neomycin on the anaerobic microflora which hydrolyses the vicine to its aglycon divicine and consequently the toxic and lethal effect was established. The present study demonstrated that the increase doses of vicine resulted in an increase in the percentage of mortality in rats; this effect may be attributed to the decrease in the concentration of GSH in blood which is associated with increase in mortality. Similar results were also obtained by Arbid and Marquardt [34]. They reported that ip administration of vicine glycoside and its subsequent hydrolytic product in the gastrointestinal tract above the critical level required to produce a lethal effect. Pretreatment with the poorly absorbed broad spectrum neomycin reduced the percentage of mortality caused by the vicine doses. This result may be attributed to the reduction of the hydrolysis of vicine and consequently the depletion of GSH. This result was in agreement with that obtained by Arbid et al. [33] where their observations provide for the first time direct evidence that hydrolysis of the glycosides by the microorganisms in the gastrointestinal tract occurred before this compound becomes toxic.

Concerning the effect on some blood parameters, the present investigation revealed that Hb concentration, Hct value, and RBCs count were significantly decreased after the experimental injection of vicine to male albino rats. The reduction in the three blood parameters may be attributed to the hemotoxicity of vicine which resulted in the premature removal of damaged red cells by the spleen [35]. This reduction is also reported by Yannai and Marquardt [36]. Regarding the effect of the neomycin, the results showed increase in the hematological parameters in the rats pretreated by neomycin and this may be due to its bactericidal effect on the microorganisms which hydrolyzes vicine to its toxic aglycon divicine. 

Concerning the effect on white blood cells count, the present results showed a decrease in WBCs count in vicine group which may be attributed mainly to the decrease in lymphocytes as a sign of direct action of the glycoside vicine on the lymphatic tissue or a sign of a continuation of the depressing effect of corticosteroids upon mitosis in the lymphatic tissue as reported by Kaneko [37]. The reduction in WBCs count was observed after injection of vicine in male albino rats was also reported by Yannai and Marquardt [36] and Arbid and Marquardt [11]. With respect to the rats pretreated by neomycin, the results showed an increase in WBCs count compared to control rats; this may be due to the decrease of hydrolysis of the glycoside to its toxic divicine [33]. 

The decrease in the level of protein and albumin may be explained by the fact that toxic liver injury is usually associated with decreased albumin level secondary to decreased protein synthesis and decrease in globulin level due to deteriorated hepatic activity as reported by Comporti [38]. The decline in serum albumin concentration was also recorded by Abbady [39] who attributed this decline in the level of serum albumin to enhanced degradation as well as enhanced loss of albumin through the gastrointestinal tract; on the other hand, neomycin treated group showed a trend almost around control group and this may be due to the effect on the microorganisms in the gastrointestinal tract and the decrease of the toxicity of the glycoside vicine. 

The significant decrease in glucose was recorded in vicine group with in accordance with Arbid and Marquardt [11]. Authors attributed the reduction in glucose level to the impairment of liver function which resulted in decrease production of glucose, altered production of glucogenic hormones including insulin, glucagons, and adrenocorticoid steroids or damage to the kidney tubules resulting in reducing the recovery of glucose from blood during glomerular filtration [40]. The pretreatment with the antibiotic neomycin modulated the oxidative stress caused by injection of vicine was due to decrease its hydrolysis to its toxic aglycon divicine and the level of glucose increased to reach the normal range.

The decrease in the level of GSH and increase in the level of lipid peroxidation and product malondialdehyde (MDA) or thiobarbituric acid reactive substance (TBARs) in serum in the groups injected by vicine are due to the production of free radicals which did not react only with erythrocytes but also with other membranes and tissues to cause tissue damage and loss of functional properties [41]. 

The present study reported a highly significant decrease in the activity of serum G6-PD in vicine group as compared to control group; this reduction may be attributed to a compensatory response to oxidative stress where the consumption of G6-PD enzyme was to maintain sufficient levels of NADPH in response to the oxidative stress [42]. All the effects of vicine on GSH, TBARs, and G6-PD rendered to almost normal value in the group treated with neomycin which decreased the toxic effect of vicine by preventing its hydrolysis to its aglycon divicine [33]. 

With regard to serum levels of liver enzymes, data of the present work showed that serum AST and ALT were significantly decreased and liver AST and ALT consequently increased due to accumulation in the liver tissue in vicine treated group. The decrease in the levels of serum AST and ALT that was observed in vicine group may be correlated with the decrease in the level of serum total protein observed in the present study as the biosynthesis of protein in vicine group was decreased. The decrease in the level of serum AST and ALT was explained by being protein in nature; these enzymes were exposed to oxidative denaturation, decreased protein synthesis by hepatic cells [43]. The increase in the activities of AST and ALT that was recorded in rats pretreated with neomycin may be attributed to its effect on the toxicity of vicine and decreasing its oxidative stress on the tissues of the rats.

The results of this study showed that pretreatment with neomycin to vicine-injected rats returned the hepatic structure almost to normal patterns and this is due to the effect of neomycin on the microorganisms in the gastrointestinal tract and the decrease of the toxicity of the glycoside vicine.

We concluded that the pretreatment with the broad spectrum antibiotic neomycin to rats injected with glycoside vicine decreased to a great extent of its toxic and mortality effects in albino rats, and this study needs further clinical investigations because this proposed model, although providing interesting results, is not representative of the clinical situation observed in G6-PD-deficient subjects after fava bean injection.

## Figures and Tables

**Table 1:** Effect of neomycin on the toxicity of vicine injected ip in rats*. *

Group	Dose (mg/kg)	No. of rats	No. of dead rats	Mortality rate %
1	0	10	0	0%
2	100	10	0	0%
3	200	10	3	30%
4	400	10	8	80%
5	250 mg/kg neomycin + 400 mg/kg vicine	10	1	10%

Group 1: control, groups 2, 3 and 4: injected vicine ip, group 5: (250 mg/kg neomycin prior to 400 mg/kg vicine).

**Table 2:** The effect of vicine and neomycin on some hematological parameters in rats.

Parameters	Group		
	Control (1 mL saline)	Vicine (400 mg/kg)	Neomycin (250 mg/kg) + Vicine (400 mg/kg)
Hb (gm/dL)	13.88 ± 0.26	9.78 ± 2.24*	12.92 ± 0.55^a^
Hct (%)	42.63 ± 1.22	30.21 ± 3.92**	39.85 ± 2.24^b^
RBCs (10^6^ cells/mm^3^)	5.82 ± 0.35	3.83 ± 0.13***	5.24 ± 0.75^b^
WBCs (10^3^ cells/mm^3^)	8.95 ± 0.55	7.85 ± 1.23	9.74 ± 2.56

Results were expressed as mean ± SD and significant difference according to control group at *P* ≤ 0.05, ANOVA showed a highly significant difference between all groups at *P* ≤ 0.0001. *P ≤ 0.05 significant difference compared to control, **P ≤ 0.01 highly significant difference compared to control. ***Very highly significant at *P* ≤ 0.001. ^a^ *P* ≤ 0.05 significant difference compared to vicine. ^b^ *P* ≤ 0.01 highly significant difference compared to vicine.

**Table 3:** Effect of vicine and neomycin on serum glucose, blood GSH, serum TBARs and serum G6-PD in rats.

Parameters	Group		
	Control (1 mL saline)	Vicine (400 mg/kg)	Neomycin (250 mg/kg) + Vicine (400 mg/kg)
Serum glucose (mg/dL)	104.51 ± 15.25	82.63 ± 6.12*	103.25 ± 14.32^a^
Blood GSH (mg/dL)	35.12 ± 1.61	21.66 ± 3.63**	31.62 ± 3.12^b^
Serum TBARs (nmol/dL)	16.22 ± 2.46	28.13 ± 3.62***	19.52 ± 3.51^b^
Serum G6-PD (U/L)	32.22 ± 3.58	17.53 ± 2.61**	29.91 ± 4.81^b^

Results were expressed as mean ± SD and significant difference according to control group at *P* ≤ 0.05, ANOVA showed a highly significant difference between all groups at *P* ≤ 0.0001. *P ≤ 0.05 significant difference compared to control, **P ≤ 0.01 highly significant difference compared to control. ***Very highly significant at *P* ≤ 0.001 compared to control. ^a^ *P* ≤ 0.05 significant difference compared to vicine. ^b^ *P* ≤ 0.01 highly significant difference compared to vicine.

**Table 4:** The effect of vicine and neomycin on serum total protein, albumin, globulin, and total protein in liver tissue in rats.

Parameters	Group		
	Control (1 mL saline)	Vicine (400 mg/kg)	Neomycin (250 mg/kg) + Vicine (400 mg/kg)
Serum total protein (g/dL)	8.57 ± 0.54	6.28 ± 0.55**	8.29 ± 0.25^b^
Serum albumin (g/dL)	5.12 ± 0.51	4.95 ± 0.82	4.86 ± 0.52
Serum globulin (g/dL)	3.45 ± 0.32	1.06 ± 0.95**	3.43 ± 0.65^b^
Liver total protein (g/g tissue)	3.25 ± 0.81	1.72 ± 0.55*	3.12 ± 0.25^a^

Results were expressed as mean ± SD and significant difference according to control group at *P* ≤ 0.05, ANOVA showed a highly significant difference between all groups at *P* ≤ 0.0001. *P ≤ 0.05 significant difference compared to control, **P ≤ 0.01 highly significant difference compared to control. ^a^ *P* ≤ 0.05 significant difference compared to vicine. ^b^ *P* ≤ 0.01 highly significant difference compared to vicine.

**Table 5:** Effect of vicine and neomycin on serum and liver transaminases (ALT and AST) in rats.

Parameters	Group		
	Control (1 mL saline)	Vicine (400 mg/kg)	Neomycin (250 mg/kg) + Vicine (400 mg/kg)
Serum AST (U/L)	17.51 ± 3.95	3.21 ± 1.23***	15.31 ± 3.51^b^
Serum ALT (U/L)	8.54 ± 3.2	5.86 ± 1.23*	8.21 ± 2.23^a^
Liver AST (U/g tissue)	48.25 ± 6.22	91.54 ± 3.23***	55.12 ± 4.5^b^
Liver ALT (U/g tissue)	35.41 ± 4.35	64.22 ± 7.51**	37.44 ± 6.25^b^

Results were expressed as mean ± SD and significant difference according to control group at *P* ≤ 0.05, ANOVA showed a highly significant difference between all groups at *P* ≤ 0.0001. *P ≤ 0.05 significant difference compared to control. **P ≤ 0.01 highly significant difference compared to control. ***Very highly significant at *P* ≤ 0.001 compared to control ^a^ *P* ≤ 0.05 significant difference compared to vicine. ^b^ *P* ≤ 0.01 highly significant difference compared to vicine.
